# UMOYA: a prospective longitudinal cohort study to evaluate novel diagnostic tools and to assess long-term impact on lung health in South African children with presumptive pulmonary TB—a study protocol

**DOI:** 10.1186/s12890-023-02329-3

**Published:** 2023-03-22

**Authors:** Isabelle Dewandel, Margaret van Niekerk, Elisabetta Ghimenton-Walters, Megan Palmer, Michaile G. Anthony, Carla McKenzie, Rolanda Croucamp, Galit Alter, Anne-Marie Demers, Gert van Zyl, Mathilda Claassen, Pierre Goussard, Ruan Swanepoel, Graeme Hoddinott, Corne Bosch, Rory Dunbar, Brian Allwood, Eric D. McCollum, H. Simon Schaaf, Anneke C. Hesseling, Marieke M. van der Zalm

**Affiliations:** 1 Desmond Tutu TB Centre, Department of Paediatrics and Child Health, Faculty of Medicine and Health Sciences, Stellenbosch University, Cape Town, South Africa; 2 Newcastle-Upon-Tyne National Health Service Hospitals Foundation Trust, Newcastle upon Tyne, UK; 3 Ragon Institute of Massachusetts General Hospital, Massachusetts Institute of Technology, and Harvard, Cambridge, MA USA; 4 Moderna Therapeutics, Cambridge, MA USA; 5 Division of Microbiology, Department of Laboratory Medicine, Centre Hospitalier Universitaire Sainte-Justine, Quebec, Canada; 6 Department of Microbiology, Immunology and Infectious Diseases, Faculty of Medicine, University of Montreal, Quebec, Canada; 7 Division of Medical Virology, Faculty of Medicine and Health Sciences, Stellenbosch University, Cape Town, South Africa; 8 National Health Laboratory Service, Tygerberg Business Unit, Cape Town, South Africa; 9 Department of Paediatric Pulmonology, Tygerberg Hospital, Stellenbosch University, Cape Town, South Africa; 10 Department of Pulmonology and Lung Function, Tygerberg Hospital, Cape Town, South Africa; 11 Department of Pulmonology, Department of Medicine, Stellenbosch University and Tygerberg Hospital, Cape Town, South Africa; 12 Global Program in Pediatric Respiratory Sciences, Eudowood Division of Pediatric Respiratory Sciences, Johns Hopkins School of Medicine, Baltimore, USA

**Keywords:** Paediatric pulmonary tuberculosis, Biomarkers, Biorepository, Lung function, Post-TB health, Lung health, Quality of life, Study protocol, Cohort, South Africa

## Abstract

**Background:**

Despite a high paediatric tuberculosis (TB) burden globally, sensitive and specific diagnostic tools are lacking. In addition, no data exist on the impact of pulmonary TB on long-term child lung health in low- and middle-income countries. The prospective observational UMOYA study aims (1) to build a state-of-the-art clinical, radiological, and biological repository of well-characterised children with presumptive pulmonary TB as a platform for future studies to explore new emerging diagnostic tools and biomarkers for early diagnosis and treatment response; and (2) to investigate the short and long-term impact of pulmonary TB on lung health and quality of life in children.

**Methods:**

We will recruit up to 600 children (0–13 years) with presumptive pulmonary TB and 100 healthy controls. Recruitment started in November 2017 and is expected to continue until May 2023. Sputum and non-sputum-based samples are collected at enrolment and during follow-up in TB cases and symptomatic controls. TB treatment is started by routine care services. Intensive follow-up for 6 months will allow for TB cases to retrospectively be classified according to international consensus clinical case definitions for TB. Long-term follow-up, including imaging, comprehensive assessment of lung function and quality of life questionnaires, are done yearly up to 4 years after recruitment.

**Discussion:**

The UMOYA study will provide a unique platform to evaluate new emerging diagnostic tools and biomarkers for early diagnosis and treatment response and to investigate long-term outcomes of pulmonary TB and other respiratory events on lung health in children.

## Background

Despite the increased awareness of the burden of paediatric TB globally over the last decades, sensitive and specific diagnostic tools are still lacking. According to the 2022 World Health Organization (WHO) global TB report, an estimated 1.2 million children developed TB in 2021. The diagnostic gap is the highest in young children, with only approximately a third of children with TB 0–5 years of age accessing care and being reported in national TB programmes [1, 2]. The gap between TB-burden estimates and reporting to WHO is due to shortfalls in detection, diagnosis, and reporting in the younger age group. Due to challenges of sample collection and the paucibacillary nature of TB disease in children, most children, especially those under 5 years of age, are clinically diagnosed. *Mycobacterium tuberculosis* (*M.tb*) culture is considered the reference standard for TB diagnosis; however the sensitivity of mycobacterial culture yield to confirm TB in children is usually between 20 and 40% [3, 4], with sputum smear microscopy for acid-fast bacilli (AFB) being positive in only 10% to 15% [5]. The roll-out of molecular testing, Xpert MTB/RIF (Cepheid, Sunnyvale, CA, USA) and more recently Xpert MTB/RIF Ultra, which initially looked promising for children with paucibacillary disease, still shows sub-optimal sensitivity [6], however specificity for both is high, herewith meeting the WHO target product profile for confirmatory diagnostic tests [7, 8]. Alternative specimen collection methods for children are nasopharyngeal aspirate (NPA), urine, saliva, and stool [9–11]. Although the collection and testing of multiple samples may contribute to an increased diagnostic yield and improved diagnosis, the implementation at primary care level, where the need for adequate diagnostic tools is the highest, remains challenging [12, 13].

### Diagnostic tools

New diagnostic tools are needed to bridge the diagnostic gap, especially in children 0–5 years of age. Host-based biomarkers, such as tuberculin skin test (TST) and interferon-gamma release assay (IGRA), have been available for decades, but have the limitation of not being able to differentiate between TB infection and disease. Currently there are novel diagnostic tests in the pipeline, including different host ribonucleic acid (RNA) gene expression signatures, which may have the potential to distinguish between TB infection and disease, but data in children are limited [14]. Similarly, algorithms can play a role in improving the diagnostic and treatment gap in childhood TB [15, 16]. Recently the WHO conditionally approved the use of a new TB diagnostic algorithm, which has 85% sensitivity and specificity of 37% with chest X-ray (CXR) features or specificity of 30% without CXR features [7, 17]. However, this algorithm still requires validation in different settings and levels of care. In addition, even though the algorithm can be used with CXR, implementing CXR imaging will require resources and training for correct interpretation. Computer-aided detection (CAD) has the potential to automate CXR reading and improve efficiency, but data in children are currently lacking [18, 19]. Point-of-care (POC) chest ultrasound has been explored as a tool for pulmonary TB diagnosis, but more data on diagnostic value and implementation are needed [20–22].

### Lung health in children with TB and other respiratory illnesses

A recent modelling study by Dodd and colleagues estimated that 155 million TB survivors were alive in 2020, of which an estimated 6–10% were children under 15 years of age [23]. Despite microbiological cure and treatment completion, data from adult TB survivors suggest a high prevalence of chronic respiratory symptoms and pulmonary impairment after TB [24, 25]. Despite the high burden of paediatric TB, data are limited on the impact of TB on long-term child lung health. Because lungs continue to develop from in utero until early adulthood, with the first years of life being the most critical for lung development, children could be potentially at high risk for post-TB lung disease (PTLD) [26–28]. A recent cross-sectional study from Gambia shows a more than threefold increase in lung function impairment in children with a history of previous TB compared to age-matched controls without a history of TB [29]. There is evidence that lower respiratory tract infections (LRTIs) during infancy reduce lung function in early childhood, which is independent of premorbid lung function [30–32]. Of the common respiratory viruses associated with LRTIs, adenovirus (AdV) and respiratory syncytial virus (RSV) have been most frequently linked to subsequent respiratory sequelae, including bronchiectasis and bronchiolitis obliterans [33–36]. However, this is especially under-researched in low- and middle-income countries (LMICs), where coincident viral co-infections in children with TB and HIV (human immunodeficiency virus) may synergistically impact on lung pathology and lung function. HIV infected and HIV exposed infants with LRTIs experience a higher incidence of hospitalisation and worse in-hospital outcomes compared to HIV unexposed children; however long-term data is lacking [37–39]. In addition, lung function impairment in children may affect their quality of life (QoL) and educational potential [40, 41]. Data on long-term outcomes in children with viral LRTIs and/or TB living in LMICs are needed to identify children at high risk for lung function impairment later in life, and the potential impact on various other aspects of their health.

## Study aims

The UMOYA study aims to: (1) build a clinical, radiological, and biological sample repository to evaluate new emerging diagnostic tools and biomarkers for early TB diagnosis and treatment response in children; (2) investigate the short and long-term impact of pulmonary TB and other respiratory illnesses on lung health and QoL in these children.

## Methods

### Study setting and population

The drug-susceptible TB case notification rate in the Western Cape Province has been steadily declining since 2008, from 950/100.000 in 2008 to 600/100.000 population in 2017 [42, 43]. More recently, in 2019, over 1.4 million children under 5 years of age received TB symptom screening in facilities of the Western Cape province, of which 0.4% had TB symptoms (defined as the presence of one or more of TB symptoms as indicated in the screening tool [44]); compared to over 6.3 million children 5 years and older, of which 3.1% were symptomatic [45]. The estimated prevalence of HIV infection amongst children with bacteriologically-confirmed TB has declined due to effective prevention of mother to child transmission (PMTCT) programmes, 12.9% of children tested before the Coronavirus disease (COVID-19) era from prospective surveillance in Cape Town [46] and 8.6% during the COVID-19 pandemic [47]. Young children with presumed TB are mainly referred to hospitals for standard diagnostic investigations. In our setting, approximately 30% of children initiating TB treatment in hospital are culture positive [3, 11]. There are no standard guidelines on monitoring TB treatment response; standard of care mainly consists of clinical follow-up and monitoring for TB treatment completion with additional imaging and/or bacteriology as clinically indicated.

In the UMOYA study, children aged 0–13 years are recruited from Tygerberg Hospital (TBH) and Karl Bremer Hospital (KBH). The two participating hospitals serve as regional referral centres to the surrounding health sub-districts. TBH also serves as a tertiary level referral centre for complicated paediatric TB. Jointly, the estimated paediatric TB caseload is approximately 600 children per year [48]. The population consists mainly of mixed-race and black African inhabitants with a large proportion living in poverty [49], over-crowded living conditions and informal housing. 

### Study design and timelines

UMOYA is an ongoing prospective observational cohort study of children presenting with presumptive pulmonary TB. Study participants are eligible if they meet the inclusion criteria and none of the exclusion criteria, as summarised in Table 1. Recruitment started in November 2017 and is expected to continue until May 2023. We will recruit up to 600 children with presumptive pulmonary TB and 100 healthy controls. Healthy controls are siblings or child household members without any signs or symptoms of pulmonary TB. At enrolment, a minimum of two respiratory samples (gastric aspirate (GA), expectorated (ES) or induced sputum (IS)) are sent to Tygerberg Hospital's nationally accredited TB laboratory (National Health Laboratory Service, NHLS) for TB microbiology testing. The attending clinician decides on whether the child receives TB treatment (TB case) or not (symptomatic control), based on the available investigation results. TB treatment is given within clinical care services according to National TB programme guidelines. In addition, minimally invasive samples are collected for storage and future analysis, and NPAs are stored for analysis of viral respiratory co-infections.

**Table 1:** Inclusion and exclusion criteria for study enrolment

Inclusion criteria	Exclusion criteria
Children 0–13 years, > 2.5 kg, living with or without HIV	Extra-thoracic TB only TB treatment for > 2 days in previous 2 weeks Alternative diagnosis at baseline Severe illness resulting in unstable clinical condition
Written informed consent from the parent or legal guardian	Informed consent not obtained Contra-indication to sampling procedures Discharge before baseline sampling completed Unstable social circumstances Residence in remote areas
Presumed pulmonary TB, inpatient or outpatient, identified in hospital with ≥ 1 of: 1. Persistent unremitting cough or wheezing of > 2 weeks, unresponsive to a course of antibiotics 2. Poor growth over the preceding 3 months 3. Persistent unexplained lethargy or reduced playfulness/activity > 2 weeks 4. Persistent unexplained fever > 1 week 5. Neonatal pneumonia, hepatosplenomegaly, or sepsis-like illness, unexplained and unresponsive to appropriate treatment 6. Any duration of cough, wheeze or acute pneumonia with ≥ 1: a. Close infectious TB contact OR b. Reactive tuberculin skin test OR c. Chest radiograph suggestive of TB 7. Recurrent (≥ 1 episode per month) respiratory symptoms	

HIV: Human immunodeficiency virus; TB: Tuberculosis

**Fig. 1:**
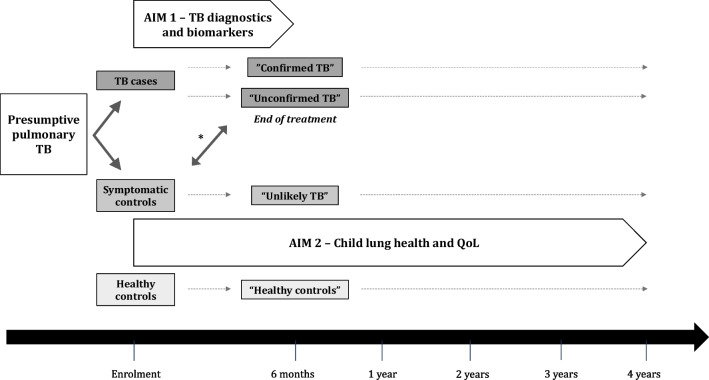
Flow diagram of study participants. Flow diagram of children enrolled in the study and followed for 4 years. *Children initially enrolled as symptomatic control are clinically checked throughout the study and if there is a clinical suspicion of tuberculosis can move to the TB cases group. TB: Tuberculosis; QoL: Quality of life

Children are followed up intensely for 6 months, with visits at baseline and at 2, 8, 16 and 24 weeks after enrolment. This intensive follow-up period allows for careful clinical evaluation of these children, for the collection of additional samples and for the retrospective classification using international consensus clinical case definitions for TB (Fig. 1) [50]. Long-term follow-up is done in all children 6 monthly up to 4 years after recruitment to establish a cohort of children with pulmonary TB and other respiratory illnesses and to investigate the long-term impact of these respiratory events on lung health in childhood.

### Expected outcomes

The primary outcome of this study is to build a state-of-the-art clinical, radiological, and biological sample repository for future studies to explore new emerging diagnostic tools and biomarkers for early diagnosis and treatment response and long-term outcomes of pulmonary TB in children. The secondary outcome is to better understand the long-term consequences of pulmonary TB and other respiratory illnesses on lung health in children. We will estimate the risk of PTLD, characterize the spectrum of PTLD, and identify children at risk for poor lung health outcomes after TB treatment. We will evaluate clinical, radiological, functional and QoL measures of lung health in well-defined children with pulmonary TB, in symptomatic controls, and healthy controls. Finally, we will characterize lung function trajectories in these different groups of interest (TB cases, symptomatic controls, and healthy controls).

### Sample size

Previous studies have shown that an estimated 50% of children will be classified as TB cases (Walters, preliminary data) and the other 50% will be symptomatic controls. The TB confirmation rate amongst bacteriologically-confirmed TB cases is expected to be 30–50%, with the remainder of the TB cases being unconfirmed cases, based on clinical grounds and response to TB treatment. There was no sample size calculation done for the primary outcome. For the secondary outcome, sample size calculation was undertaken to detect differences in lung function outcomes between TB cases and healthy controls*.* We hypothesize that children with pulmonary TB will have a forced expiratory volume in 1 s (FEV1) that is 0.5 z-score lower compared to healthy controls. To achieve 90% power at a significance level of p < 0.05, the recruitment target will be adequately powered with an estimated total of 600 children with presumed TB, allowing for a 20% loss to follow-up.

### Study investigations

Clinical and respiratory assessments, biological sample collections, and collections of health-related quality of life (HRQoL) and socio-economic data are performed according to the schedule of events (SOE) (Tables 2 and 3) using standard operating procedures. The study investigations are divided to investigate the 2 main aims; diagnostic (first 6 months) and lung health (up until 4 years after enrolment).

**Table 2:** Schedule of Events (SOE)—Diagnostics study phase

	Baseline	Week 2	Week 8	Week 16	Week 24	Unscheduled visit
History and clinical review	X	X	X	X	X	X
Mantoux	X^a^		X^b^			(X)
Chest radiography	X^a^				X^a^	(X)
Chest US^c^	X^d^		X^d^			
TB smear, liquid culture (MGIT) and Xpert MTB/RIF Ultra^e^	X^e^	X^e^	X^e^	X^e^	X^e^	(X)
Serum for TB biomarkers^f,g^	X	X^d^	X^d^	X^d^	X	(X)
Whole blood (PAXgene RNA tube) for TB biomarkers^f,g^	X					(X)
Urine for TB biomarkers^f^	X	X^d^	X^d^		X^d^	(X)
Stool sample^f^	X					(X)
Saliva sample^f^	X				X^d^	(X)
NPA sample for viral detection and storage^f^	X	X	X		X	(X)

RNA: Ribonucleic acid; MGIT: Mycobacteria Growth Indicator Tube; NPA: Nasopharyngeal aspirate; (X) If applicable

^a^Healthy control once and thereafter if clinically indicated

^b^If baseline Mantoux negative

^c^Chest ultrasound (US) in a specific subgroup

^d^Tuberculosis (TB) cases and symptomatic controls;

^e^Two respiratory samples—only in TB cases and symptomatic controls; smear and culture (not Xpert MTB/RIF Ultra) only if positive culture at week 2 until the culture is negative

^f^Storage in biorepository

^g^Maximum blood volumes per age are respected as per internationally accepted and National Institutes of Health (NIH) research guidelines

**Table 3:** Schedule of Events (SOE)—Lung health study phase

	Week 24	Week 52	Week 104	Week 156	Week 208
History and clinical review	X	X	X	X	X
Quality of life questionnaires (TANDI, EDQ-5DY)	X	X	X	X	X
ISAACS and respiratory symptoms questionnaire	X	X	X	X	X
Chest radiography^a^	X	X	X	X	X
Lung function testing					
Spirometry > 4 years of age	X	X	X	X	X
TremoFlo oscillometry > 2 years	X	X	X	X	X
Diffusion capacity > 7 years of age				X	
Plethysmography > 7 years of age				X	
6-min walk test > 4 years of age			X	X	

^a^Healthy control once and if clinically indicated this is repeated

#### Diagnostics study phase

After the baseline visit, follow-up visits are done at weeks 2, 8, 16 and 24. Data collected include questionnaires, TB diagnostic samples, imaging, and lung function assessments in children with presumptive pulmonary TB. For the healthy controls, certain baseline investigations are done to allow for a comparison group; for detailed investigations see Table 2.

##### Questionnaires and clinical assessment

Baseline and follow-up questionnaires collect detailed clinical information relevant for TB diagnosis and risk factors for respiratory illnesses. A detailed history of presenting symptoms (including TB contact exposure), previous medical history (including vaccinations—Bacille Calmette-Guérin (BCG) and other—and previous hospital admissions for respiratory illnesses), environmental exposure (smoking and biomass fuel), an adapted version of the International Study of Asthma and Allergies in Childhood (ISAACS) [51], and a shortened version of the St. Georges respiratory questionnaire are collected in all children [52]. Physical examination includes assessment of nutritional status including anthropometry, general physical assessment, and in-depth respiratory assessment. Children living with HIV are staged according to the WHO clinical stages ranging from stage 1—asymptomatic—to stage 4—acquired immunodeficiency syndrome (AIDS).

##### TB diagnostics

Comprehensive routine TB diagnostic sampling procedures include TST and *M.tb* microbiology on a minimum of 2 respiratory samples (GA, ES or IS). Concentrated smear microscopy (Auramine O), culture in BACTEC Mycobacteria Growth Indicator Tube 960 (MGIT 960) liquid medium (Becton Dickinson, MD), and Xpert MTB/RIF or Ultra (Ultra from 28 March 2018) are done on all respiratory samples. If the culture results positive, drug susceptibility testing (DST) is done using a line-probe assay (GenoType MTBDR*plus*; Hain Lifescience, Nehren, Germany).

##### Radiological assessment

All children have CXR at presentation, and at follow-up as per SOE (Tables 2 and 3). For retrospective TB case classification, all CXRs are being reviewed independently by 2 paediatric TB expert CXR readers to determine diagnostic certainty and severity of TB disease as per clinical case definitions and latest WHO operational handbook [7]. Images are de-identified and electronically stored in an image biorepository for expert reading and potential CAD reading. Information on additional imaging is collected if done as part of clinical care—including computerized tomography (CT) scan of the chest, and bronchoscopy.

##### Additional sample collection and biorepository

NPAs are collected at enrollment and follow-up as per SOE for viral detection in all children. The commercially available multiplex polymerase chain reaction (PCR) (Anyplex™ II, RV16, Seegene) respiratory panel is completed in the Tygerberg Medical Virology Laboratory. The panel includes 16 viruses of clinical and epidemiological relevance: AdV, influenza A/B virus, parainfluenza virus 1–4, human rhinovirus (HRV), RSV, bocavirus, metapneumovirus, coronavirus 229E, NL63, OC43 and enterovirus. In addition, severe acute respiratory syndrome coronavirus-2 (SARS-CoV-2) PCR is done in all samples collected after March 2020. For the biorepository, we store blood (serum and whole blood collected in PAXgene blood RNA tube), stool, urine, NPA and saliva from the enrolled children as per SOE. In addition, positive *M.tb* cultured isolates are stored for future use and genotyping.

#### Lung health study phase

Lung function measurements are done 6, 12, 24, 36 and 48 months after initial enrollment, depending on the age of the child and feasibility of these measurements.

An additional telephonic questionnaire is used between the yearly visits to limit reporting bias between visits and to support retention. For detailed investigations see Table 3.

##### Questionnaires

Questionnaires collect information about respiratory events and health care visits after enrolment. We record reported acute or chronic respiratory symptoms, intercurrent chest infections, clinic visits, new episodes of TB exposure or TB disease, medication use (including bronchodilators, antibiotics, and steroids) and hospitalisation.

##### Quality of life assessment

Standardized QoL questionnaires are used to assess the impact of respiratory illnesses on the QoL of these children and their caregivers. HRQoL questionnaires include the use of the Toddler and infant questionnaire (TANDI) [53] and the European QoL-5 dimension for youth questionnaire (EQ-5D-Y) [54]. The use of caregiver proxy versions of the QoL questionnaires allows for caregivers to report on behalf of young children, additionally, self-reported versions are implemented for older children.

##### Imaging

CXR is done 6, 12, 24, 36 and 48 months after enrollment in both TB cases and symptomatic controls or if clinically indicated in healthy controls. Dual expert reading focuses on chronic radiological changes by comparing the CXRs over time.

##### Lung function and functional lung health measurements

Lung function measurements are done according to feasibility, depending on measurement and age, at any of the study visits from enrollment until 4 years later.

Lung function measurements include oscillometry, spirometry, plethysmography, and diffusion capacity. Pre- and post-bronchodilation are done for both oscillometry and spirometry measurements. Functional assessment is done using the 6-min walk test (6MWT).

A. TremoFlo airwave oscillometry

Oscillometry is a tidal breathing technique that can be done in children from the age of 2 years. TremoFlo (©ThoraSys, Canada) adds an oscillatory wave to regular, quiet breathing and measures lung resistance (Rrs) and reactance (Xrs). The intra-breath measurements of respiratory rate and tidal volume reflect changes within the breathing cycle and may be more sensitive for detecting early lung disease [55].

B. Spirometry

Spirometry is a forced maneuver that can be done in children from the age of 4 years. The spirometry tests (Jaeger Full Masterscreen PFT. Germany—2011. CD version 5.72.1.77) are completed in a sitting position according to European Respiratory Society (ERS)/American Thoracic Society (ATS) recommendations, with an inline bacterial filter and prediction equations from the Global Lung Initiative (GLI) 2012 [56, 57]. The spirometry system is calibrated with a 3L calibration syringe daily and routinely disinfected. During the test procedure, the participant initially breathes normally through the mouthpiece. Participants are then instructed to inspire maximally followed by an immediate, forced exhalation, continuing to complete exhalation. Once an expiratory plateau is reached, defined as a less than 25 ml change in volume for at least one second, the child is instructed to inhale maximally again. The *post-bronchodilator response* is completed by comparing flow volume curves before and 15–20 min after 400ug of a short-acting bronchodilator (salbutamol), administered via a spacer.

C. Single-breath diffusion test

The diffusion test (Jaeger) can usually be done in children from the age of 7 years. The child breathes 4–5 tidal breaths, is then asked to exhale maximally followed by a maximal, rapid inspiration to total lung capacity (TLC). During inspiration, the child inhales 0.3% acetylene (C_2_H_2_), 0.3% carbon monoxide (CO), 21% oxygen (O_2_), 0.3% methane (CH_4_) and a balance of nitrogen (N_2_). Breath-hold lasts for 10 s at TLC, followed by a rapid exhalation. During exhalation, the expired gas is analyzed to measure lung diffusion capacity for carbon monoxide (DL_CO_). Gas analysis (sample volume) is taken within 4 s of expiration, after breath hold. DL_CO_ indicates the transfer rate of CO in the air of the lung into the pulmonary capillary blood [58, 59].

D. Plethysmography

Plethysmography (Jaeger) is done in children from the age of 7 years. Children are seated within a transparent “box”. Initially the child breathes rapidly, and airway resistance (Raw) is determined. When the shutter is closed, mouth pressure is assumed to be equal to alveolar pressure. When the shutter opens, the child breathes to TLC and exhales to residual volume (RV) [60].

E. The 6-min walk test (6MWT)

The 6MWT can be done in children from the age of 4. This is a standardised test, which quantifies functional exercise capacity by letting the participant walk for 6 min at their own pace. The baseline and end-of-test heart rate and oxygen saturation (SpO_2_) are measured using a pulse-oximeter. The lung function technologist completes this test using standard protocols and ATS guidelines [61, 62].

## Data management

Study data are collected and managed using REDCap (Research Electronic Data Capture), web-based software platform developed by Vanderbilt University and supported by the REDCap Consortium. The electronic data capture tool is hosted at Stellenbosch University [63, 64]. REDCap is a secure, web-based software platform designed to support data capture for research studies, providing (1) an intuitive interface for validated data capture; (2) audit trails for tracking data manipulation and export procedures; (3) automated export procedures for seamless data downloads to common statistical packages, and (4) procedures for data integration and interoperability with external sources. The server environment, which hosts the database, is secure within the network of Stellenbosch University and governed by standard medical information security guidelines, which includes the Health Insurance Portability and Accountability Act of 1996 (Public Law 104–191), also known as HIPAA. Data sharing complies with ethical and data rights, including intellectual property considerations. Data sharing is addressed and protected through data transfer agreements and participants are informed through the informed consent process. The study adheres to the Protection of Personal Information Act (POPI Act, No. 4 of 2013). All personal information (names and contact information) collected, is stored in a secure and access-controlled location. Identifiable data are stored separately to the main database. Personal identifiers are removed from all datasets shared according to the clinical quality management plan from the Desmond Tutu TB centre and as instructed by the Stellenbosch University research data management regulations [65].

We will use STROBE guidelines for reporting observational research.

## Study monitoring

Monitoring of study activities and data collection are carried out by a central on-site team comprised of a study coordinator, data manager and study clinician. Study coordinator and data manager are responsible for the query management. Implementation training was undertaken at study initiation. Regular updates and refresher training sessions are provided to clinical and operational study staff to ensure correct and efficient application of all study procedures.

## Ethical considerations

The study is conducted according to South African and internationally accepted ethical standards, including the Declaration of Helsinki and the South African Good Clinical Practice guidelines [66]. Study protocol, Informed consent form (ICF) and other relevant study documents have been approved by the relevant Ethics Committees. An annual progress report is submitted to the Ethics committee and funders. Prior to enrolment, the parents/legal caregivers of study participants provide written informed consent, including children > 7 years who need to provide written informed assent. At recruitment, each patient is assigned a unique anonymous study identifier. All data are stored without personal identifiers on protected servers with limited access. The study was approved by the local Health Research Ethics Committee of Stellenbosch University (N17/08/083).

## Discussion

Current diagnostic approaches in childhood TB perform poorly, especially in young children and children living with HIV. The lack of reliable diagnostics for paediatric TB contributes to the wide diagnostic gap between children having TB disease and children being diagnosed and reported with TB disease, and likely contributes to a significant proportion of preventable TB deaths in these vulnerable children. There is an urgent need for better tools to diagnose TB, especially in young children and those living with HIV. This unique, well-characterized paediatric cohort, with a wide spectrum of disease provides an optimal opportunity to build a state-of-the-art clinical, radiological, and biological sample repository for future discovery and validation of diagnostic tools in pulmonary TB.

In addition, the long-term follow-up of this well-characterized cohort allows to investigate the impact of pulmonary TB and other respiratory illnesses on long-term child lung health in LMICs like South Africa. These findings will allow for increased awareness and advocacy for child lung health and the future development of preventive and tailored therapeutic strategies in children at risk for poor outcomes, including tailored anti-TB treatment approaches, targeted vaccination for viruses such as influenza, RSV or SARS-CoV-2; and immune-modulating antibiotics.

In conclusion, improving TB diagnostics and providing new insights in the long-term morbidity of pulmonary TB in children could contribute to filling gaps in the TB care cascade, including defining important outcome indicators for childhood TB in the future.

## Dissemination of results

We anticipate that the results of this study will be relevant to a broader research audience. Moreover, we will disseminate the findings of this study through different channels, such as scientific articles in international peer-reviewed journals and presentations at national and international conferences. The dissemination of the research results will be an integral part of the UMOYA study, which also focuses on strengthening of collaboration through developing new and intensifying existing links between the involved research institutions for exchange of knowledge; and motivate for additional collaboration with other research networks in the future.

## Data Availability

The datasets used and/or analysed during the current study are available from the corresponding author on reasonable request.

## Abbreviations

TB: Tuberculosis
WHO: World Health Organization
*M.tb*: Mycobacterial tuberculosis
AFB: Acid-fast bacilli
NPA: Nasopharyngeal aspirate
TST: Tuberculin skin test
IGRA: Interferon-gamma release assay
RNA: Ribonucleic acid
CXR: Chest X-ray
CAD: Computer-aided detection
POC: Point-of-care
PTLD: Post-tuberculosis lung disease
LRTIs: Lower respiratory tract infections
AdV: Adenovirus
RSV: Respiratory syncytial virus
LMICs: Low- and middle-income countries
HIV: Human immunodeficiency virus
QoL: Quality of life
PMTCT: Prevention of mother to child transmission
COVID-19: Coronavirus disease
TBH: Tygerberg Hospital
KBH: Karl Bremer Hospital
GA: Gastric aspirate
ES: Expectorated sputum
IS: Induced sputum
FEV1: Forced expiratory volume in 1 s
HRQoL: Health-related quality of life
SOE: Schedule of events
BCG: Bacille Calmette-Guérin
ISAACS: International Study of Asthma and Allergies in Childhood
AIDS: Acquired immunodeficiency syndrome
MGIT: Mycobacteria Growth Indicator Tube
DST: Drug susceptibility testing
CT: Computerized tomography
PCR: Polymerase chain reaction
HRV: Human rhinovirus
SARS-CoV-2: Severe acute respiratory syndrome coronavirus-2
TANDI: Toddler and infant questionnaire
EQ-5D-Y: European QoL-5 dimension for youth questionnaire
6MWT: 6-Minute walk test
Rrs: Resistance
Xrs: Reactance
ERS: European Respiratory Society
ATS: American Thoracic Society
GLI: Global Lung Initiative
TLC: Total lung capacity
C2H2: Acetylene
CO: Carbon monoxide
O2: Oxygen
CH4: Methane
N2: Nitrogen
DLCO: Diffusion capacity for carbon monoxide
Raw: Airway resistance
RV: Residual volume
SpO2: Oxygen saturation
REDCap: Research Electronic Data Capture
ICF: Informed consent form
